# Rapid evolution of the *env* gene leader sequence in cats naturally infected with feline immunodeficiency virus

**DOI:** 10.1099/vir.0.000035

**Published:** 2015-04

**Authors:** Paweł M. Bęczkowski, Joseph Hughes, Roman Biek, Annette Litster, Brian J. Willett, Margaret J. Hosie

**Affiliations:** 1 MRC University of Glasgow Centre for Virus Research, University of Glasgow, Glasgow, UK; 2 Small Animal Hospital, University of Glasgow, Glasgow, UK; 3 Boyd Orr Centre for Population and Ecosystem Health & Institute of Biodiversity, Animal Health & Comparative Medicine, University of Glasgow, Glasgow, UK; 4 Department of Veterinary Clinical Sciences, Purdue University, West Lafayette, IN 47907, USA

## Abstract

Analysing the evolution of feline immunodeficiency virus (FIV) at the intra-host level is important in order to address whether the diversity and composition of viral quasispecies affect disease progression. We examined the intra-host diversity and the evolutionary rates of the entire *env* and structural fragments of the *env* sequences obtained from sequential blood samples in 43 naturally infected domestic cats that displayed different clinical outcomes. We observed in the majority of cats that FIV *env* showed very low levels of intra-host diversity. We estimated that *env* evolved at a rate of 1.16×10^−3^ substitutions per site per year and demonstrated that recombinant sequences evolved faster than non-recombinant sequences. It was evident that the V3–V5 fragment of FIV *env* displayed higher evolutionary rates in healthy cats than in those with terminal illness. Our study provided the first evidence that the leader sequence of *env*, rather than the V3–V5 sequence, had the highest intra-host diversity and the highest evolutionary rate of all *env* fragments, consistent with this region being under a strong selective pressure for genetic variation. Overall, FIV *env* displayed relatively low intra-host diversity and evolved slowly in naturally infected cats. The maximum evolutionary rate was observed in the leader sequence of *env*. Although genetic stability is not necessarily a prerequisite for clinical stability, the higher genetic stability of FIV compared with human immunodeficiency virus might explain why many naturally infected cats do not progress rapidly to AIDS.

## Introduction

Deciphering the evolution of feline immunodeficiency virus (FIV) is essential for understanding why some infected cats have normal lifespans, whilst others progress rapidly to AIDS. The generation of polymorphic populations (Eigen, 1993) and the potentially high intra-host diversity of RNA viruses are features attributable to their biology as well as the unique characteristics of retroviral reverse transcription (Domingo, 2000). Improved understanding of the dynamics of viral populations at the intra-host level will lead to the development of more effective diagnostic tests and control strategies, and ultimately, will inform the design of the next generation of FIV vaccines, as the limited efficacy of the current commercial vaccine (Dunham *et al.*, 2006) is likely attributable to the high genetic diversity of naturally occurring viruses.

The majority of FIV phylogenetic analyses have focused on sequence variation at the population level (Carpenter *et al.*, 1998; Olmsted *et al.*, 1992; Pistello *et al.*, 1997; Samman *et al.*, 2011; Teixeira *et al.*, 2010) and highlighted the high variability of the V3–V5 region of the *env* gene, which has been used to classify FIV into six distinct subtypes (A–E and putative subtype F) (Marçola *et al.*, 2013; Sodora *et al.*, 1994). Employing gard (Kosakovsky Pond *et al.*, 2006a) and jpHMM (Schultz *et al.*, 2006) recombination detection methods, we recently observed an abundance of recombinant *env* sequences in natural FIV infection, emphasizing the important role of recombination in generating viral diversity at the population level and highlighting the limitations of the current phylogenetic classification of FIV (Bęczkowski *et al.*, 2014a).

Mutations also contribute to viral diversity, although little is known about the evolutionary rate and the within-host viral diversity of the entire FIV *env* in natural infection (Table 1). In human immunodeficiency virus type 1 (HIV-1)-infected individuals, quasispecies composition varies greatly, with some studies reporting 10 % diversity in long-term infected individuals (Delwart *et al.*, 1997). It has also been proposed that intra-host diversity could influence the outcome of disease and that the rate of viral evolution decreases during disease progression (Delwart *et al.*, 1997; Shankarappa *et al.*, 1999).

**Table 1.** Comparison of evolutionary rates of the *env* gene of HIV-1 and fragments of the *env* of FIV-Fca (FIV strain of domestic cat) and FIV-Pco (FIV strain of cougar)

Virus	Evolutionary rate [×10^−3^ substitutions per site per year (95% HPD)]	Study
HIV-1 gp160 *env*	2.4 (1.8–2.8)	Korber *et al.* (2000)
FIV-Fca V3–V6 *env*	3.1–6.6 (2.2–4.1, 5.0–8.4, 3.3–9.2)*	Hayward & Rodrigo (2010)
FIV-Pco V4–V5 *env*	1.54 (0.89–2.23)	Biek *et al.* (2003)
FIV-Fca V1–V2 *env*	3.4†	Greene *et al.* (1993)
FIV-Fca V1–V9 *env*	0.9‡ (0.18–1.59)–6.7§ (1.72–13.35)	Kraase *et al.* (2010)

* 95 % HPD for each of three cats (Hayward & Rodrigo, 2010).

† Evolutionary rate estimated using method of Gojobori and Yokoyama (Greene *et al.*, 1993).

‡ At 322 weeks post-infection.

§ At 12 weeks post-infection (Kraase *et al.*, 2010).

Selection measures have been applied previously to assess forces acting on the evolution of different retroviral genes and at different regions within the same gene (Choisy *et al.*, 2004). For example, in HIV-1 infection, *gag* is under negative selection pressure (Kils-Hütten *et al.*, 2001), whilst *env* evolution is generally shaped by positive selection pressure (Wolfs *et al.*, 1990). As *gag* encodes structural proteins, its conserved nature is essential to maintain viral integrity. In contrast, HIV-1 *env* encodes a heavily glycosylated envelope glycoprotein in which mutations in *N*-linked glycosylation sites are important for immune evasion (Yamaguchi & Gojobori, 1997). By analogy with HIV, we predict that FIV *env* is under a similar positive selection pressure as its human counterpart, but relatively little is known about the glycosylation pattern or selection forces acting on FIV Env in naturally infected cats.

To our knowledge, there are no data available concerning rates of evolution of the entire ORF of FIV *env* which, in contrast to primate retroviruses, contains an unusually long leader/signal region (Verschoor *et al.*, 1993). Evolutionary studies of HIV-1 infection (Korber *et al.*, 2000; Leitner & Albert, 1999; Lukashov *et al.*, 1995; Shankarappa *et al.*, 1999; Zhang *et al.*, 1997) suggested that within-host evolution is influenced by both viral- and host-dependent factors. Whereas some studies reported increased evolutionary rates during the early stages of infection, declining towards terminal stage disease (Delwart *et al.*, 1997; Shankarappa *et al.*, 1999), others did not observe a similar pattern (Mikhail *et al.*, 2005). Nevertheless, the assumption that the virus is under constant pressure in immunocompetent individuals and evolves at a high rate in response to a changing environment provides a plausible explanation for differences in evolutionary rates at different stages of disease (Lukashov *et al.*, 1995). It is apparent that rates of evolution are dependent on the combined effects of host and viral factors, demonstrated by comparisons of the evolutionary tempo of different HIV-1 subtypes (Abecasis *et al.*, 2009), and differences in replication rates of HIV-1 and -2 (MacNeil *et al.*, 2007b).

Our ability to understand immune evasion, and to predict the outcome of FIV infection, depends on understanding how the intra-host evolution of FIV differs within and among cats. For this reason, we examined *env* sequences collected serially over 12 months from two distinct cohorts of naturally infected cats that developed different clinical outcomes. Our specific aims were to (i) quantify viral diversity, selection and rates of evolution in naturally infected cats, and (ii) determine whether the evolutionary rate correlated with the clinical outcomes of FIV infection.

## Results

### Phylogenetic inference

**Fig. 1.**
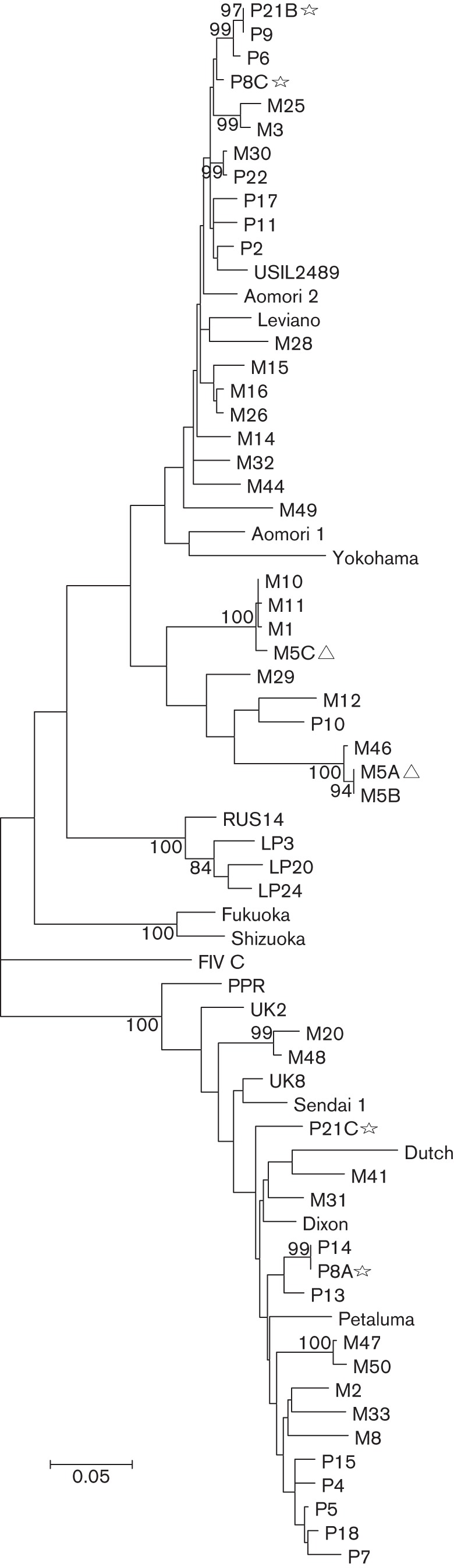
ML tree, based on the HKY model, rooted on a clade C reference FIV *env*. The tree is drawn to scale, with branch lengths measured in the number of substitutions per site. The ML phylogeny includes 47 entire *env* nucleotide sequences (representative of a total of 355 sequences from Chicago and Memphis), 15 entire *env* sequences derived from GenBank: Aomori 1 (GenBank accession number D37816), Aomori 2 (GenBank accession number D37817.1), FIV C (GenBank accession number AF474246.1), Dixon (GenBank accession number L00608.1), Dutch (GenBank accession number X60725), Fukuoka (GenBank accession number D37815.1), Sendai 1 (GenBank accession number D37813.1), Shizuoka (GenBank accession number D37811.1), UK2 (GenBank accession number X69494.1), UK8 (GenBank accession number X69496.1), USIL2489 (GenBank accession number U11820.1), Yokohama (GenBank accession number D37812.1), Petaluma (GenBank accession number M25381.1), PPR (GenBank accession number M36968.1), Leviano (GenBank accession number FJ374696.1), three V3–V5 region sequences representing clade E: LP3 (GenBank accession number D84496), LP20 (GenBank accession number D84498) and LP24 (GenBank accession number D84500), and one shorter 504 bp in length RUS14 (GenBank accession number EF447297) sequence. Taxa with inconsistent clade assignment are represented with a star (P8, P21). Non-monophyletic taxa from cat M5 are marked with a triangle. Only bootstrap values >80 are shown.

The maximum-likelihood (ML) tree constructed using the entire dataset (Additional file 1, available in the online Supplementary Material) revealed high genetic diversity in FIV *env* at the population level (overall mean pairwise distance of 14.1 %). Although >41 % of cats were infected with circulating recombinant viruses, we found no evidence of within-host recombination (Bęczkowski *et al.*, 2014a). At the intra-host level, sequential sequences amplified from 93 % (40/43) of cats clustered together in monophyletic groups, whilst sequences from three cats (M5, P8 and P21), amplified at different time points, showed incongruent phylogenetic assignment (Fig. 1). Sequences from a parent/offspring pair (cat M1 was an offspring of female M11) were classified as non-recombinant clade B and clustered closely together, with an overall mean pairwise distance of 0.3 % (Fig. S1).

### Intra-host diversity

**Fig. 2.**
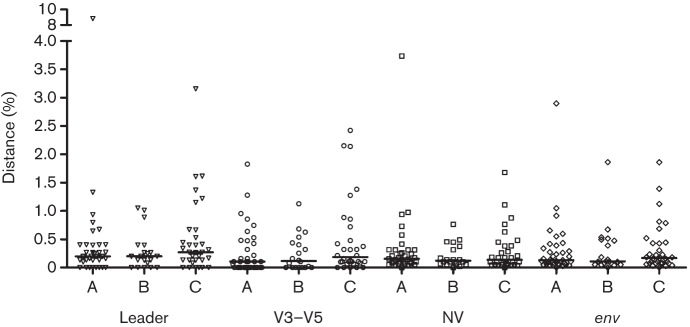
Comparison of mean pairwise distances between *env* fragments and entire *env* sequences amplified from three samplings (A, B and C on the *x*-axis). Median values for the leader, V3–V5, NV and *env*, respectively, for time points A (0.20, 0.11, 0.16 and 0.13 %), B (0.20, 0.12, 0.12 and 0.11 %) and C (0.27, 0.19, 0.14 and 0.17 %).

Having excluded sequences from the three cats that formed non-monophyletic groups, the mean pairwise distances for *env* within cats ranged from 0 to 2.9 % (median 0.13 %), 0 to 1.9 % (median 0.11 %) and 0 to 1.9 % (median 0.17 %), for early (A), intermediate (B) and late (C) time points, respectively (Table S1). When we compared the diversity of structural fragments of *env*, although not statistically significant (*P* = 0.29), the leader region displayed the highest variation at the intra-host level; the median overall mean pairwise distances for each time point were 0.2, 0.2 and 0.27 %, respectively (Fig. 2, Table S1).

### Selection

**Fig. 3.**
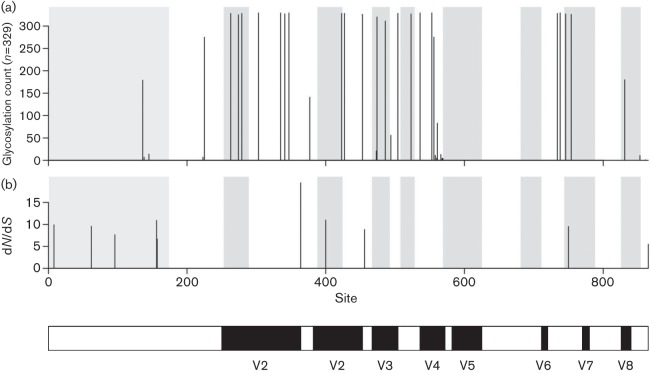
Frequency and location of predicted PNGSs (a) and sites under positive selection (b) with corresponding d*N*/d*S* (ω) values. PNGSs at positions 303, 335, 347, 536, 553 and 738 represent fixed sequons among all clades of FIV from different geographical origins (*n* = 329). Sequons at positions 263, 279, 423, 427, 504, 734 and 341, 523 and 746 were also fixed, and were present in >99 % of sequences. Positively selected sites (*n* = 10) were consistently identified by three different detection methods (for details, see Table S2). Leader sequence and B-cell epitope regions identified in previous studies are highlighted in grey. The putative leader sequence cleavage site is located at the position 528 bp from the start codon of the *env* ORF of reference FIV Petaluma (GenBank accession number M25381.1). Location of V1–V8 regions is highlighted below the graphs.

The mean ω value (d*N*/d*S* ratio) determined by SLAC (single likelihood ancestor counting) for the *env* sequences from both cohorts was 0.34. Examining the entire dataset, the SLAC analysis indicated 14 (1.6 %) positively and 201 (23 %) negatively selected sites. FEL (fixed effects likelihood) reported 22 (2.5 %) positively and 391 (44.8 %) negatively selected sites, whilst IFEL (internal fixed effects likelihood) indicated 24 (2.7 %) and 377 (43.2 %) sites under positive and negative selection, respectively. Positively selected sites, consistently identified by the three methods (Table S2), were restricted to specific regions of *env*, with a high proportion (5/10, 50 %; sites 8, 62, 96, 156 and 157) lying within the leader region (Fig. 3). The selection hot spots at positions 400 and 750 were located within B-cell epitopes identified in previous studies (Lombardi *et al.*, 1993; Pancino *et al.*, 1993) (Fig. 3). An investigation of the global distribution of potential *N*-linked glycosylation sites (PNGSs) for the presence of fixed and shifting sequons revealed that, regardless of the high genetic variability of the analysed sequences (*n* = 329), conserved PNGSs did exist (Fig. 3). However, none of the predicted PNGSs fell within sites identified as being under positive selection.

### Rates of molecular evolution

**Fig. 4.**
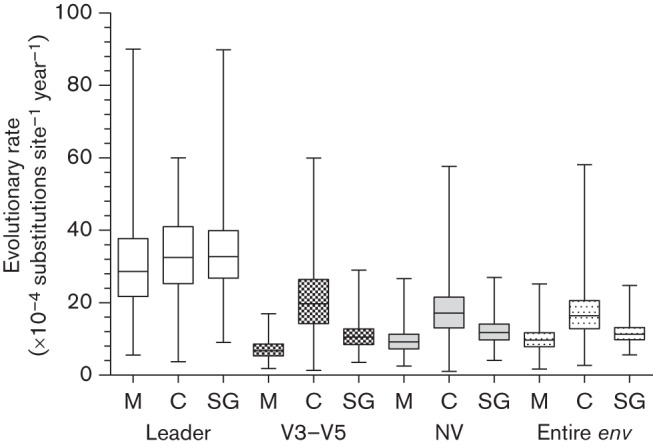
Distribution of the evolutionary rates calculated for the leader, V3–V5 fragment, NV fragment and entire *env* gene. Evolutionary rates are compared between sequences amplified from Memphis (M), Chicago (C) and all cats (SG). Rate estimates were either based on strict or relaxed clock models (for details, see Table S3).

For all cats in the study, *env* was estimated to evolve at a mean rate of 1.16×10^−3^ [0.7–1.67 95 % highest posterior density (HPD)] substitutions per site per year under a relaxed log-normal clock model (Table S3) with the first and second codon positions evolving at a lower rate than the third codon position [0.95 (0.90–0.98 95 % HPD) versus 1.1 (1.02–1.19 95 % HPD)]. To determine whether specific regions of the *env* gene evolved at different rates, we estimated the clock rate for the leader, the V3–V5 and the non-variable (NV) regions. The leader region evolved at the highest rate [3.43×10^−3^ (1.55–5.6 95 % HPD) substitutions per site per year under a relaxed log-normal clock model] in comparison with other fragments, whilst the evolutionary rate of the V3–V5 region, commonly regarded as highly variable, was over three times lower [1.08×10^−3^ (0.5–1.74 95 % HPD) substitutions per site per year under a relaxed log-normal clock model] (Fig. 4, Table S3).

**Fig. 5.**
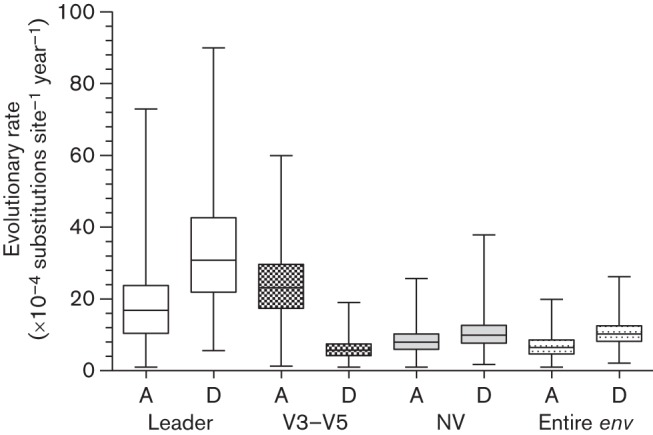
Comparison of evolutionary rates of the leader, V3–V5, NV fragments and the entire *env* genes from six alive (healthy, A) and 11 deceased (unhealthy, D) cats from the Memphis cohort during the 12 month observation period. Rate estimates were either based on strict or relaxed clock models (for details, see Table S3).

As the individually housed cats from the Chicago cohort were generally healthy, whilst the majority of group housed cats from the Memphis cohort had high morbidity and 63 % mortality over the study period, we compared the evolutionary rates between and within the cohorts. Median rates of evolution of the entire *env* and its fragments were higher for the sequences from cats in the Chicago cohort (Fig. 4). We hypothesized that *env* evolution in healthy cats that remained alive throughout the study period would be faster than in terminally ill cats that died during the study period. Fig. 5 illustrates a comparison of the evolutionary rates for sequences from (i) 11 deceased cats, and (ii) six cats that remained alive and generally free of clinical signs during the study. The entire *env*, leader and NV fragment evolved at higher rates in terminally ill animals. This relationship, however, was reversed for the V3–V5 region, which evolved at a higher rate in cats that remained alive during the study period. Recombinant *env* sequences (*n* = 123) evolved three times faster than sequences without any detectable prior history of recombination (*n* = 184) (Table S3).

## Discussion

Analyses of serial *env* sequences collected over 12 months from naturally infected cats offered a rare opportunity to examine the intra-host dynamics and evolution of FIV. Despite considerable variation among individuals, the overall intra-host diversity of *env* (up to 3 %) was low compared with primate immunodeficiency viruses (commonly >5 %) (Rambaut *et al.*, 2004; Salemi, 2013). Furthermore, the overall mean distance between sequences obtained from two closely related animals (M1 was the offspring of M11), following at least one postulated transmission event, was remarkably low. These results suggest that the *env* sequences were stably maintained and evolved slowly following vertical transmission. This is consistent with an earlier study examining V3–V5 *env* sequence diversity of the Aomori 2 strain of FIV following vertical transmission; 100 % homology was observed between viruses isolated from a queen and her kitten 48 weeks post-transmission (Motokawa *et al.*, 2005).

The apparent discrepancy between low, within-host viral diversity and relatively high diversity of FIV at the population level is intriguing, especially in light of differences in evolutionary rates demonstrated at the within-host and epidemiological levels in HIV infection (Alizon & Fraser, 2013; Lythgoe & Fraser, 2012). Analysis of FIV evolutionary rates at the epidemiological level are constrained by the low number of longitudinal full-length FIV *env* sequences that are available and comprehensive datasets would be needed to quantify these reliably.

Low diversity and rates of evolution in lentiviruses are commonly associated with lower pathogenicity. Although HIV-1 and -2 achieve similar proviral loads in infected individuals, HIV-2 replicates to lower titres in the plasma (Popper *et al.*, 2000), and displays a lower rate of sequence evolution (MacNeil *et al.*, 2007a) and lower pathogenicity, reflected by a slower decline in CD4^+^ T-cell numbers and slower disease progression (Marlink *et al.*, 1988, 1994; Popper *et al.*, 1999; Whittle *et al.*, 1994). Similarly, bovine immunodeficiency virus, which has low pathogenicity, is more closely related to FIV than to primate lentiviruses (Olmsted *et al.*, 1989) and also exhibits very little sequence variation (Carpenter *et al.*, 2000).

The genetic stability of some lentiviruses could be associated with the fidelity of their reverse transcriptase (Lewis *et al.*, 1999). Mutations in the catalytic YMDD motif of HIV-1 and the V148S mutation in simian immunodeficiency virus (SIV) (Huisman *et al.*, 2008) have been implicated in increasing reverse transcriptase fidelity (Olmsted *et al.*, 1989; Operario *et al.*, 2005). It is likely that the reverse transcriptase of FIV has enhanced fidelity compared with the reverse transcriptase of HIV-1 (Huisman *et al.*, 2008). The presence of almost identical sequences in our study might further indicate that FIV exhibits a low replication rate during persistent infection or that most infections occur via cell-to-cell transfer owing to feline tetherin/BST-2 (Dietrich *et al.*, 2011). It is also possible that viral evolution is constrained due to the high fitness cost associated with divergence from the parental virus (Domingo & Holland, 1997). Whatever the specific mechanism, the low sequence variation of FIV in comparison with HIV-1 could be indicative of co-adaptation between FIV and its host, which are thought to have co-existed together for longer than HIV-1 and humans (Pecon-Slattery *et al.*, 2008). Furthermore, the analysis of viral sequence divergence and disease pathogenesis amongst the Felidae suggests different periods of virus–host co-evolution (Troyer *et al.*, 2008). In general, the least pathogenic viruses appear to have co-evolved with their hosts for longer periods of time, whereas more virulent strains have more recent origins. This pattern is reflected in the disease-inducing potential of different species-specific FIVs, with FIV-Pco (FIV of the puma) being the least pathogenic, whilst FIV-Fca (FIV of the domestic cat) is the most pathogenic. Although FIV infection in pumas and lions was previously reported to be asymptomatic, recent evidence suggests that infected lions do indeed exhibit mild clinical sings of immunodeficiency, subsequently affecting the lifespans of infected animals (Roelke *et al.*, 2009). Similar observations have been made recently in SIV-infected chimpanzees, previously regarded as asymptomatic virus carriers (Terio *et al.*, 2011).

It has been suggested that the slow rate of evolution documented in terminal HIV-1 infection might be linked to the weakened host immune system (Delwart *et al.*, 1997; Shankarappa *et al.*, 1999). Consistent with this theory, we found that the V3–V5 fragment, which contains neutralization epitopes and is under immune system surveillance (Lombardi *et al.*, 1993; Pancino *et al.*, 1993), evolved faster in healthy cats than in terminally ill cats. In addition, the rates of evolution across all datasets from the individually housed cats from Chicago (which were generally in good health) were consistently higher than those estimated for sequences from the cats from Memphis (which displayed more clinical signs of illness).

Recombination is a potential driver of rapid evolutionary change and plays a significant role in generating viral diversity among retroviruses (Onafuwa-Nuga & Telesnitsky, 2009). In the present study, viruses with a prior history of recombination (Bęczkowski *et al.*, 2014a) exhibited higher evolutionary rates, which could be the result of strong immune selection pressure acting on the recombinant Envs compared with (presumably) more host-adapted, evolutionary older non-recombinants. Nonetheless, the mechanisms responsible for the faster evolution of recombinants might not be related to the immune system, and could be attributed to their higher replication tempo and increased infectivity (Tebit *et al.*, 2007).

This is the first study to demonstrate that against a background of low diversity and slow evolution for *env* as a whole, the leader region exhibited the highest proportion of positively selected sites and the highest evolutionary rate among all fragments examined, but more data are required to confirm this. The N-terminal signal peptide of FIV plays an important role in post-translational targeting of the Env precursor and its translocation through the endoplasmic reticulum (Verschoor *et al.*, 1993). Studies of viral leader sequences highlighted their enormous complexity, suggesting a role not only in post-translational events, but also in post-cleavage events (Hegde & Bernstein, 2006; Lemberg & Martoglio, 2002). Leader-encoded peptides are involved in self-antigen presentation (Borrego *et al.*, 1998) – a property exploited by human cytomegalovirus. This virus has a signal peptide with nine residues identical to the MHC class I signal peptides (Tomasec *et al.*, 2000), which helps it to evade detection by NK cells presenting a virus-encoded MHC class I molecule (Ulbrecht *et al.*, 2000). Furthermore, sequence variation among the signalling peptides of acute and chronic isolates of HIV has also been demonstrated (Gnanakaran *et al.*, 2011). These findings suggest that the leader may play an important role in regulating *env* expression on the viral surface (Hegde & Bernstein, 2006) and therefore viral infectivity. This hypothesis warrants further research and validation in functional studies.

By comparing amino acid sequences and global patterns of PNGSs, 19 sites were identified which were conserved in >97 % of the examined sequences, with six PNGSs displaying 100 % fixed cross-clade pattern. This striking consistency at the population level suggests that glycosylation at these sites is likely to play an essential role in the folding and integrity of the viral Env. Patterns of fixed *N*-linked glycosylation sites have also been noted in infections with SIV (Chackerian *et al.*, 1997) and HIV-1 group M subtypes A–G (Gao *et al.*, 1996).

Statistical characterization of a relationship between within-host evolutionary dynamics and the clinical outcome of retroviral infection remains problematic. Although HPDs permit a comparison of evolutionary rates between groups of patients, the estimate uncertainty tends to be high due to limited signal within intra-host datasets (Carvajal-Rodríguez *et al.*, 2008). We have considered hierarchical phylogenetic models to test for rate differences between cats, but preliminary analyses suggested limited power to detect effects in our data using these parameter-rich models, as also suggested by others (Edo-Matas *et al.*, 2011).

The conclusions drawn from the intra-host diversity and rates of evolution are restricted by (i) the 63 % mortality rate within the Memphis cohort, (ii) the relatively low number of sequences available from some cats at selected time points and (iii) the short 12-month study period (Seo *et al.*, 2002), and indeed we identified almost identical sequences over the three different sampling times. However, despite this short time frame and the potential limitations of the methodology used, we reliably quantified within-host evolution. The presence of identical amplicons in cats at three different sampling times suggest that factors which tend to generate false diversity (polymerase template switching, PCR-induced errors) did not affect our results. Furthermore, the results presented here are in agreement with previous FIV studies employing ‘bulk’ PCR (Ikeda *et al.*, 2004; Motokawa *et al.*, 2005), end-point dilution proviral DNA PCR (Kraase *et al.*, 2010) as well as a study examining sequences from plasma viral RNA (Huisman *et al.*, 2008), and we observed low sequence variation and high genetic stability of FIV in a relatively large sample of naturally infected cats.

## Conclusion

We observed relatively low intra-host diversity and a low rate of evolution of the entire *env* in cats naturally infected with FIV. The greater overall genetic stability of FIV compared with HIV-1 might explain why many naturally infected cats do not progress rapidly to AIDS. To the best of our knowledge, this is the first study to demonstrate that the leader sequence is the fastest evolving region of *env*. As the majority of previous studies focused on the V3–V5 region, these results indicate that the role of the unusually long leader sequence of FIV *env* in viral infectivity and immune evasion might have been underestimated.

## Methods

### 

#### Cats, FIV *env* sequences and alignments.

In total, 44 privately owned, neutered domestic cats, of various ages, breeds and conditions of health, were enrolled into the study (Bęczkowski, 2013) based on a history of a positive FIV antibody test result (SNAP FIV/FeLV Combo Test; IDEXX Laboratories). All cats were feline leukemia virus antigen-negative and their FIV-positive status was confirmed by virus isolation (Hosie *et al.*, 2009). In the study group, 27 cats lived together in a large multi-cat household in Memphis, TN, USA, where FIV-positive and FIV-negative cats were housed indoors with unrestricted access to one another. The remaining 17 cats lived in individual households in Chicago, IL, USA, with exception of five cats: two cats (P7 and P4) had been rehomed together and were living in the same household, one cat (P9) had been rehomed with another FIV-positive cat not enrolled in the study, one cat (P13) had been rehomed with another FIV-negative cat, and one cat (P21) was housed with another two FIV-positive cats in the rehoming centre. Cats were classified, based on a 6-monthly clinical examination by a registered specialist in feline medicine (A. L.), into two groups: (i) healthy (cats with no abnormalities found on clinical examination) and (ii) unhealthy (cats with any abnormalities detected on clinical examination). At the time of enrolment, there were 10 healthy (59 %) and seven unhealthy (41 %) cats in the Chicago cohort. In the Memphis cohort, 12 cats were classified as healthy (44 %) and 15 cats were classified as unhealthy (56 %) at the time of enrolment. During the study period, there was a 63 % mortality rate in the Memphis cohort (17/27), whilst only one cat (5.9 %) from the Chicago cohort died during the same time frame (Bęczkowski, 2013).

Multiple full-length FIV *env* genes (~2500 bp) were amplified directly from whole blood collected at 6-monthly intervals starting in January and May 2010 for Memphis and Chicago, respectively (time points A, B and C), using a nested PCR protocol (Table S4). First-round PCR products were amplified directly from blood, without genomic DNA extraction by Phusion Blood Direct II Polymerase (Thermo Fisher Scientific) followed by direct nucleic acid sequence determination. Phusion Blood Direct II Polymerase is a proofreading polymerase with 25-fold greater accuracy than *Taq* DNA polymerase, determined with a modified *lac*I-based method (Frey & Suppmann, 1995). The nucleic acid sequence of the first-round PCR product informed the primer design for the second-round PCR, which was performed using High Fidelity PCR Master (Roche), which amplifies with threefold greater accuracy than *Taq* DNA polymerase (Roche). Strain-specific primers for the second-round PCR incorporated restriction sites. Amplified *env* sequences were cloned into the eukaryotic expression vector VR1012 (Hartikka *et al.*, 1996) and transformed into *Escherichia coli* MAX Efficiency DH5α Competent Cells (Invitrogen). Thus constructed VR1012 plasmids expressing FIV *env* were sequenced (Table S5) using a Big Dye Terminator v1.1 kit (Applied Biosystems) for the purpose of the present study, before being assessed in functional studies (Bęczkowski *et al.*, 2014b, c). Special measures were taken to avoid the possibility of contamination, in both the clinical and laboratory settings: cats were double identified prior to blood sampling, PCRs were prepared in a designated UV-treated room and fresh, unopened reagents were used at each separate time point throughout the study.

There were 355 serial *env* sequences from 43 cats available for analysis from the two cohorts (Table S5). The number of sequences varied according to the availability of follow-up samples, being largely influenced by the 63 % mortality rate within the Memphis cohort. Multiple sequence alignments were conducted in mega5 (Tamura *et al.*, 2011) and curated manually to ensure homology of gaps in sequences of variable length. Analyses were performed using the entire *env* DNA sequences and the three structural fragments: (i) leader/signal region (~509 bp in length), (ii) variable V3–V5 region (~630 bp) and (iii) remaining concatenated fragments of the entire *env* after exclusion of the V3–V5 region, denoted NV (~1900 bp). The temporal signal and ‘clocklikeness’ of the *env* phylogenies were tested in Path-o-Gen (http://tree.bio.ed.ac.uk) (Table S6).

#### Phylogenetic trees.

ML trees were constructed in mega5 (Tamura *et al.*, 2011) under the HKY nucleotide substitution model, selected through jMODELTEST analysis (Posada, 2008). Statistical support for ML trees was estimated using bootstrapping analysis with 1000 replicates (Efron *et al.*, 1996). The ML tree constructed using the entire dataset was carefully examined for evidence of non-monophyletic clustering of multiple sequences amplified from each individual. Sequences from three animals (M5, P8 and P21) amplified at different time points did not form monophyletic groups, and were excluded from further intra-host and evolutionary rate analyses.

#### Intra-host diversity.

Intra-host sequence variation among the *env* sequences and three fragments of the gene (leader, V3–V5 and NV) at each time point were calculated as mean and highest pairwise distances under the HKY nucleotide substitution model in paup 4.0 v10 (Wilgenbusch & Swofford, 2002). To explore the variation in mean pairwise distances of three structural fragments of *env*, a general linear model (Khan, 2013) was used with ‘structural fragment of the *env*’ and ‘time point’ as fixed effects and ‘cat’ as a random effect. No evidence of recombination within hosts was found using rigorous fivefold recombination testing (Bęczkowski *et al.*, 2014a).

#### Selection and PNGSs.

Nucleotide sites under diversifying or purifying selection were identified and d*N*/d*S* ratios (ω) for every codon in the alignment were estimated using three different methods: SLAC (Kosakovsky Pond & Frost, 2005) at *P*<0.01, FEL (Kosakovsky Pond & Frost, 2005) at *P*<0.1 and IFEL (Kosakovsky Pond *et al.*, 2006b) at *P*<0.1. To focus on the most strongly supported positively selected sites, we reported those sites which were consistently identified by all three methods.

PNGSs and their position in the protein alignment were identified by the *N*-GlycoSite tool available at the Los Alamos National Laboratory web server (http://www.hiv.lanl.gov/content/index) and the number of sequences with glycosylation sites was counted. The analysis included reference sequences from GenBank representing clades A–D: Aomori 1 (GenBank accession number D37816), Aomori 2 (GenBank accession number D37817.1), FIV C (GenBank accession number AF474246.1), Dixon (GenBank accession number L00608.1), Dutch (GenBank accession number X60725), Fukuoka (GenBank accession number D37815.1), Sendai 1 (GenBank accession number D37813.1), Shizuoka (GenBank accession number D37811.1), UK2 (GenBank accession number X69494.1), UK8 (GenBank accession number X69496.1), USIL2489 (GenBank accession number U11820.1), Yokohama (GenBank accession number D37812.1), Petaluma (GenBank accession number M25381.1), PPR (GenBank accession number M36968.1) and sequences from all cats excluding identical, duplicate sequences (*n* = 329). Alignments were numbered according to positions in the M49C C76 sequence.

#### Rate of molecular evolution.

Rates of evolution of the entire *env* gene and individual fragments from each Memphis and Chicago cohorts and all cats were estimated using beast v1.7.1 (Drummond & Rambaut, 2007), based on sampling date information. Additionally, rate comparisons were made between non-recombinant or recombinant sequences from healthy and sick cats, and cats from Memphis that remained alive and those that died during the study period.

Sequence alignments from animals from which data were available from more than one time point were included in the analysis: 17 cats from Memphis (197 sequences) and 12 cats from Chicago (108 sequences). The analysis of differences in evolutionary rate between sequences from alive (*n* = 6) and deceased cats (*n* = 11) was based on sequences from Memphis cohort. The HKY evolutionary model of substitution with four-category Γ distribution was selected with codon positions (1+2) and 3 as partitions (Shapiro *et al.*, 2006). The analyses in beast were performed estimating independent trees for each cat, with a linked clock rate parameter across individual cats, under strict and relaxed log-normal clock models with uniform distribution clock rate priors, informed by previous estimates of evolutionary rates for FIV and HIV (Table 1). The length of the Markov chain Monte Carlo was set for 2×10^8^ iterations with 2×10^7^ burn-in, and was run until convergence and effective sample sizes >100 were obtained.

The beast-generated output log file was analysed in tracer v1.5. The coefficient of variation in rates among branches was used to determine whether a relaxed molecular clock was more appropriate for these data. This was assumed to be the case if the estimate for the coefficient of variation excluded zero.

Statistical support was provided in the form of parameter estimates and their HPD values.

#### Ethics and consent.

The study and its aims were reviewed and approved by the University of Glasgow Ethics Committee and the Purdue Animal Care and Use Committee. Cat owners provided written informed consent for their participation in the study.

## References

[r1] Abecasis A. B. Vandamme A. M. Lemey P. **(**2009**).** Quantifying differences in the tempo of human immunodeficiency virus type 1 subtype evolution. J Virol 83, 12917–12924. 10.1128/JVI.01022-09 19793809 PMC2786833

[r2] Alizon S. Fraser C. **(**2013**).** Within-host and between-host evolutionary rates across the HIV-1 genome. Retrovirology 10, 49. 10.1186/1742-4690-10-49 23639104 PMC3685529

[r3] Bęczkowski P. M. **(**2013**).** Virus evolution in the progression of natural feline immunodeficiency virus infection. PhD thesis, Centre for Virus Research, University of Glasgow, UK.

[r4] Bęczkowski P. M. Hughes J. Biek R. Litster A. Willett B. J. Hosie M. J. **(**2014a**).** Feline immunodeficiency virus (FIV) *env* recombinants are common in natural infections. Retrovirology 11, 80 10.1186/s12977-014-0080-1 25699660 PMC4180853

[r5] Bęczkowski P. M. Techakriengkrai N. Logan N. McMonagle E. Litster A. Willett B. J. Hosie M. J. **(**2014b**).** Emergence of CD134 cysteine-rich domain 2 (CRD2)-independent strains of feline immunodeficiency virus (FIV) is associated with disease progression in naturally infected cats. Retrovirology 11, 95. 10.1186/s12977-014-0095-7 25430586 PMC4275942

[r6] Bęczkowski P. M. Logan N. McMonagle E. Litster A. Willett B. J. Hosie M. J. **(**2014c**).** An investigation of the breadth of neutralising antibody response in cats naturally infected with feline immunodeficiency virus. J Gen Virol 10.1099/vir.0.071522-0 [Epub ahead of print]. 10.1099/vir.0.071522-0 25395594 PMC4336861

[r7] Biek R. Rodrigo A. G. Holley D. Drummond A. Anderson C. R. Jr Ross H. A. Poss M. **(**2003**).** Epidemiology, genetic diversity, and evolution of endemic feline immunodeficiency virus in a population of wild cougars. J Virol 77, 9578–9589. 10.1128/JVI.77.17.9578-9589.2003 12915571 PMC187433

[r8] Borrego F. Ulbrecht M. Weiss E. H. Coligan J. E. Brooks A. G. **(**1998**).** Recognition of human histocompatibility leukocyte antigen (HLA)-E complexed with HLA class I signal sequence-derived peptides by CD94/NKG2 confers protection from natural killer cell-mediated lysis. J Exp Med 187, 813–818. 10.1084/jem.187.5.813 9480992 PMC2212178

[r9] Carpenter M. A. Brown E. W. MacDonald D. W. O’Brien S. J. **(**1998**).** Phylogeographic patterns of feline immunodeficiency virus genetic diversity in the domestic cat. Virology 251, 234–243. 10.1006/viro.1998.9402 9837787

[r10] Carpenter S. Vaughn E. M. Yang J. Baccam P. Roth J. A. Wannemuehler Y. **(**2000**).** Antigenic and genetic stability of bovine immunodeficiency virus during long-term persistence in cattle experimentally infected with the BIV(R29) isolate. J Gen Virol 81, 1463–1472. 10811930 10.1099/0022-1317-81-6-1463

[r11] Carvajal-Rodríguez A. Posada D. Pérez-Losada M. Keller E. Abrams E. J. Viscidi R. P. Crandall K. A. **(**2008**).** Disease progression and evolution of the HIV-1 *env* gene in 24 infected infants. Infect Genet Evol 8, 110–120. 10.1016/j.meegid.2007.10.009 18249158

[r12] Chackerian B. Rudensey L. M. Overbaugh J. **(**1997**).** Specific *N*-linked and *O*-linked glycosylation modifications in the envelope V1 domain of simian immunodeficiency virus variants that evolve in the host alter recognition by neutralizing antibodies. J Virol 71, 7719–7727. 9311856 10.1128/jvi.71.10.7719-7727.1997 PMC192123

[r13] Choisy M. Woelk C. H. Guégan J.-F. Robertson D. L. **(**2004**).** Comparative study of adaptive molecular evolution in different human immunodeficiency virus groups and subtypes. J Virol 78, 1962–1970. 10.1128/JVI.78.4.1962-1970.2004 14747561 PMC369455

[r14] Delwart E. L. Pan H. Sheppard H. W. Wolpert D. Neumann A. U. Korber B. Mullins J. I. **(**1997**).** Slower evolution of human immunodeficiency virus type 1 quasispecies during progression to AIDS. J Virol 71, 7498–7508. 9311829 10.1128/jvi.71.10.7498-7508.1997 PMC192096

[r15] Dietrich I. McMonagle E. L. Petit S. J. Vijayakrishnan S. Logan N. Chan C. N. Towers G. J. Hosie M. J. Willett B. J. **(**2011**).** Feline tetherin efficiently restricts release of feline immunodeficiency virus but not spreading of infection. J Virol 85, 5840–5852. 10.1128/JVI.00071-11 21490095 PMC3126296

[r16] Domingo E. **(**2000**).** Viruses at the edge of adaptation. Virology 270, 251–253. 10.1006/viro.2000.0320 10792982

[r17] Domingo E. Holland J. J. **(**1997**).** RNA virus mutations and fitness for survival. Annu Rev Microbiol 51, 151–178. 10.1146/annurev.micro.51.1.151 9343347

[r18] Drummond A. J. Rambaut A. **(**2007**).** BEAST: Bayesian evolutionary analysis by sampling trees. BMC Evol Biol 7, 214. 10.1186/1471-2148-7-214 17996036 PMC2247476

[r19] Dunham S. P. Bruce J. MacKay S. Golder M. Jarrett O. Neil J. C. **(**2006**).** Limited efficacy of an inactivated feline immunodeficiency virus vaccine. Vet Rec 158, 561–562. 10.1136/vr.158.16.561 16632531

[r20] Edo-Matas D. Lemey P. Tom J. A. Serna-Bolea C. van den Blink A. E. van ’t Wout A. B. Schuitemaker H. Suchard M. A. **(**2011**).** Impact of CCR5delta32 host genetic background and disease progression on HIV-1 intrahost evolutionary processes: efficient hypothesis testing through hierarchical phylogenetic models. Mol Biol Evol 28, 1605–1616. 10.1093/molbev/msq326 21135151 PMC3080134

[r21] Efron B. Halloran E. Holmes S. **(**1996**).** Bootstrap confidence levels for phylogenetic trees. Proc Natl Acad Sci U S A 93, 13429–13434. 10.1073/pnas.93.23.13429 8917608 PMC24110

[r22] Eigen M. **(**1993**).** Viral quasispecies. Sci Am 269, 42–49. 10.1038/scientificamerican0793-42 8337597

[r23] Frey B. Suppmann B. **(**1995**).** Demonstration of the Expand™ PCR system’s greater fidelity and higher yields with a *lac*I-based fidelity assay. Biochemica 2, 34–35.

[r24] Gao F. Morrison S. G. Robertson D. L. Thornton C. L. Craig S. Karlsson G. Sodroski J. Morgado M. Galvao-Castro B. **& other authors (**1996**).** Molecular cloning and analysis of functional envelope genes from human immunodeficiency virus type 1 sequence subtypes A through G. The WHO and NIAID Networks for HIV Isolation and Characterization. J Virol 70, 1651–1667. 8627686 10.1128/jvi.70.3.1651-1667.1996 PMC189989

[r25] Gnanakaran S. Bhattacharya T. Daniels M. Keele B. F. Hraber P. T. Lapedes A. S. Shen T. Gaschen B. Krishnamoorthy M. **& other authors (**2011**).** Recurrent signature patterns in HIV-1 B clade envelope glycoproteins associated with either early or chronic infections. PLoS Pathog 7, e1002209. 10.1371/journal.ppat.1002209 21980282 PMC3182927

[r26] Greene W. K. Meers J. del Fierro G. Carnegie P. R. Robinson W. F. **(**1993**).** Extensive sequence variation of feline immunodeficiency virus *env* genes in isolates from naturally infected cats. Arch Virol 133, 51–62. 10.1007/BF01309743 8240017

[r27] Hartikka J. Sawdey M. Cornefert-Jensen F. Margalith M. Barnhart K. Nolasco M. Vahlsing H. L. Meek J. Marquet M. **& other authors (**1996**).** An improved plasmid DNA expression vector for direct injection into skeletal muscle. Hum Gene Ther 7, 1205–1217. 10.1089/hum.1996.7.10-1205 8793545

[r28] Hayward J. J. Rodrigo A. G. **(**2010**).** Molecular epidemiology of feline immunodeficiency virus in the domestic cat (*Felis catus*). Vet Immunol Immunopathol 134, 68–74. 10.1016/j.vetimm.2009.10.011 19896220 PMC2821968

[r29] Hegde R. S. Bernstein H. D. **(**2006**).** The surprising complexity of signal sequences. Trends Biochem Sci 31, 563–571. 10.1016/j.tibs.2006.08.004 16919958

[r30] Hosie M. J. Addie D. Belák S. Boucraut-Baralon C. Egberink H. Frymus T. Gruffydd-Jones T. Hartmann K. Lloret A. Lutz H. **(**2009**).** Feline immunodeficiency. ABCD guidelines on prevention and management. J Feline Med Surg 11, 575–584. 10.1016/j.jfms.2009.05.006 19481037 PMC7129779

[r31] Huisman W. Schrauwen E. J. A. Rimmelzwaan G. F. Osterhaus A. D. M. E. **(**2008**).** Intrahost evolution of envelope glycoprotein and OrfA sequences after experimental infection of cats with a molecular clone and a biological isolate of feline immunodeficiency virus. Virus Res 137, 24–32. 10.1016/j.virusres.2008.05.009 18602181

[r32] Ikeda Y. Miyazawa T. Nishimura Y. Nakamura K. Tohya Y. Mikami T. **(**2004**).** High genetic stability of TM1 and TM2 strains of subtype B feline immunodeficiency virus in long-term infection. J Vet Med Sci 66, 287–289. 10.1292/jvms.66.287 15107558

[r33] Khan R. M. **(**2013**).** Analysis of variance. In Problem Solving and Data Analysis using Minitab, pp. 150–208 New York: Wiley 10.1002/9781118307502.ch5

[r34] Kils-Hütten L. Cheynier R. Wain-Hobson S. Meyerhans A. **(**2001**).** Phylogenetic reconstruction of intrapatient evolution of human immunodeficiency virus type 1: predominance of drift and purifying selection. J Gen Virol 82, 1621–1627. 11413373 10.1099/0022-1317-82-7-1621

[r35] Korber B. Muldoon M. Theiler J. Gao F. Gupta R. Lapedes A. Hahn B. H. Wolinsky S. Bhattacharya T. **(**2000**).** Timing the ancestor of the HIV-1 pandemic strains. Science 288, 1789–1796. 10.1126/science.288.5472.1789 10846155

[r36] Kosakovsky Pond S. L. Frost S. D. **(**2005**).** Not so different after all: a comparison of methods for detecting amino acid sites under selection. Mol Biol Evol 22, 1208–1222. 10.1093/molbev/msi105 15703242

[r37] Kosakovsky Pond S. L. Posada D. Gravenor M. B. Woelk C. H. Frost S. D. **(**2006a**).** Automated phylogenetic detection of recombination using a genetic algorithm. Mol Biol Evol 23, 1891–1901. 10.1093/molbev/msl051 16818476

[r38] Kosakovsky Pond S. L. Frost S. D. W. Grossman Z. Gravenor M. B. Richman D. D. Brown A. J. L. **(**2006b**).** Adaptation to different human populations by HIV-1 revealed by codon-based analyses. PLOS Comput Biol 2, e62. 10.1371/journal.pcbi.0020062 16789820 PMC1480537

[r39] Kraase M. Sloan R. Klein D. Logan N. McMonagle L. Biek R. Willett B. J. Hosie M. J. **(**2010**).** Feline immunodeficiency virus *env* gene evolution in experimentally infected cats. Vet Immunol Immunopathol 134, 96–106. 10.1016/j.vetimm.2009.10.015 19897254

[r40] Leitner T. Albert J. **(**1999**).** The molecular clock of HIV-1 unveiled through analysis of a known transmission history. Proc Natl Acad Sci U S A 96, 10752–10757. 10.1073/pnas.96.19.10752 10485898 PMC17955

[r41] Lemberg M. K. Martoglio B. **(**2002**).** Requirements for signal peptide peptidase-catalyzed intramembrane proteolysis. Mol Cell 10, 735–744. 10.1016/S1097-2765(02)00655-X 12419218

[r42] Lewis D. A. Bebenek K. Beard W. A. Wilson S. H. Kunkel T. A. **(**1999**).** Uniquely altered DNA replication fidelity conferred by an amino acid change in the nucleotide binding pocket of human immunodeficiency virus type 1 reverse transcriptase. J Biol Chem 274, 32924–32930. 10.1074/jbc.274.46.32924 10551858

[r43] Lombardi S. Garzelli C. La Rosa C. Zaccaro L. Specter S. Malvaldi G. Tozzini F. Esposito F. Bendinelli M. **(**1993**).** Identification of a linear neutralization site within the third variable region of the feline immunodeficiency virus envelope. J Virol 67, 4742–4749. 8392611 10.1128/jvi.67.8.4742-4749.1993 PMC237860

[r44] Lukashov V. V. Kuiken C. L. Goudsmit J. **(**1995**).** Intrahost human immunodeficiency virus type 1 evolution is related to length of the immunocompetent period. J Virol 69, 6911–6916. 7474108 10.1128/jvi.69.11.6911-6916.1995 PMC189608

[r45] Lythgoe K. A. Fraser C. **(**2012**).** New insights into the evolutionary rate of HIV-1 at the within-host and epidemiological levels. Proc Biol Sci 279, 3367–3375. 10.1098/rspb.2012.0595 22593106 PMC3385732

[r46] MacNeil A. Sankale J. L. Meloni S. T. Sarr A. D. Mboup S. Kanki P. **(**2007a**).** Long-term intrapatient viral evolution during HIV-2 infection. J Infect Dis 195, 726–733. 10.1086/511308 17262716

[r47] MacNeil A. Sarr A. D. Sankalé J. L. Meloni S. T. Mboup S. Kanki P. **(**2007b**).** Direct evidence of lower viral replication rates *in vivo* in human immunodeficiency virus type 2 (HIV-2) infection than in HIV-1 infection. J Virol 81, 5325–5330. 10.1128/JVI.02625-06 17329334 PMC1900238

[r48] Marçola T. G. Gomes C. P. Silva P. A. Fernandes G. R. Paludo G. R. Pereira R. W. **(**2013**).** Identification of a novel subtype of feline immunodeficiency virus in a population of naturally infected felines in the Brazilian Federal District. Virus Genes 46, 546–550. 10.1007/s11262-013-0877-3 23329009

[r49] Marlink R. G. Ricard D. M’Boup S. Kanki P. J. Romet-Lemonne J. L. N’Doye I. Diop K. Simpson M. A. Greco F. **& other authors (**1988**).** Clinical, hematologic, and immunologic cross-sectional evaluation of individuals exposed to human immunodeficiency virus type-2 (HIV-2). AIDS Res Hum Retroviruses 4, 137–148. 10.1089/aid.1988.4.137 3259142

[r50] Marlink R. Kanki P. Thior I. Travers K. Eisen G. Siby T. Traore I. Hsieh C. C. Dia M. C. **& other authors (**1994**).** Reduced rate of disease development after HIV-2 infection as compared to HIV-1. Science 265, 1587–1590. 10.1126/science.7915856 7915856

[r51] Mikhail M. Wang B. Lemey P. Beckthold B. Vandamme A. M. Gill M. J. Saksena N. K. **(**2005**).** Role of viral evolutionary rate in HIV-1 disease progression in a linked cohort. Retrovirology 2, 41. 10.1186/1742-4690-2-41 15985187 PMC1190217

[r52] Motokawa K. Hohdatsu T. Imori A. Arai S. Koyama H. **(**2005**).** Mutations in feline immunodeficiency (FIV) virus envelope gene V3–V5 regions in FIV-infected cats. Vet Microbiol 106, 33–40. 10.1016/j.vetmic.2004.12.016 15737471

[r53] Olmsted R. A. Hirsch V. M. Purcell R. H. Johnson P. R. **(**1989**).** Nucleotide sequence analysis of feline immunodeficiency virus: genome organization and relationship to other lentiviruses. Proc Natl Acad Sci U S A 86, 8088–8092. 10.1073/pnas.86.20.8088 2813380 PMC298220

[r54] Olmsted R. A. Langley R. Roelke M. E. Goeken R. M. Adger-Johnson D. Goff J. P. Albert J. P. Packer C. Laurenson M. K. **& other authors (**1992**).** Worldwide prevalence of lentivirus infection in wild feline species: epidemiologic and phylogenetic aspects. J Virol 66, 6008–6018. 1382145 10.1128/jvi.66.10.6008-6018.1992 PMC241478

[r55] Onafuwa-Nuga A. Telesnitsky A. **(**2009**).** The remarkable frequency of human immunodeficiency virus type 1 genetic recombination. Microbiol Mol Biol Rev 73, 451–480. 10.1128/MMBR.00012-09 19721086 PMC2738136

[r56] Operario D. J. Reynolds H. M. Kim B. **(**2005**).** Comparison of DNA polymerase activities between recombinant feline immunodeficiency and leukemia virus reverse transcriptases. Virology 335, 106–121. 10.1016/j.virol.2005.02.010 15823610

[r57] Pancino G. Chappey C. Saurin W. Sonigo P. **(**1993**).** B epitopes and selection pressures in feline immunodeficiency virus envelope glycoproteins. J Virol 67, 664–672. 7678301 10.1128/jvi.67.2.664-672.1993 PMC237417

[r58] Pecon-Slattery J. Troyer J. L. Johnson W. E. O’Brien S. J. **(**2008**).** Evolution of feline immunodeficiency virus in Felidae: implications for human health and wildlife ecology. Vet Immunol Immunopathol 123, 32–44. 10.1016/j.vetimm.2008.01.010 18359092 PMC2774529

[r59] Pistello M. Cammarota G. Nicoletti E. Matteucci D. Curcio M. Del Mauro D. Bendinelli M. **(**1997**).** Analysis of the genetic diversity and phylogenetic relationship of Italian isolates of feline immunodeficiency virus indicates a high prevalence and heterogeneity of subtype B. J Gen Virol 78, 2247–2257. 9292012 10.1099/0022-1317-78-9-2247

[r60] Popper S. J. Sarr A. D. Travers K. U. Guèye-Ndiaye A. Mboup S. Essex M. E. Kanki P. J. **(**1999**).** Lower human immunodeficiency virus (HIV) type 2 viral load reflects the difference in pathogenicity of HIV-1 and HIV-2. J Infect Dis 180, 1116–1121. 10.1086/315010 10479138

[r61] Popper S. J. Sarr A. D. Guèye-Ndiaye A. Mboup S. Essex M. E. Kanki P. J. **(**2000**).** Low plasma human immunodeficiency virus type 2 viral load is independent of proviral load: low virus production *in vivo*. J Virol 74, 1554–1557. 10.1128/JVI.74.3.1554-1557.2000 10627569 PMC111493

[r62] Posada D. **(**2008**).** jModelTest: phylogenetic model averaging. Mol Biol Evol 25, 1253–1256. 10.1093/molbev/msn083 18397919

[r63] Rambaut A. Posada D. Crandall K. A. Holmes E. C. **(**2004**).** The causes and consequences of HIV evolution. Nat Rev Genet 5, 52–61. 10.1038/nrg1246 14708016

[r64] Roelke M. E. Brown M. A. Troyer J. L. Winterbach H. Winterbach C. Hemson G. Smith D. Johnson R. C. Pecon-Slattery J. **& other authors (**2009**).** Pathological manifestations of feline immunodeficiency virus (FIV) infection in wild African lions. Virology 390, 1–12. 10.1016/j.virol.2009.04.011 19464039 PMC2771374

[r65] Salemi M. **(**2013**).** The intra-host evolutionary and population dynamics of human immunodeficiency virus type 1: a phylogenetic perspective. Infect Dis Rep 5 (Suppl 1), e3. 10.4081/idr.2013.s1.e3 24470967 PMC3892624

[r66] Samman A. McMonagle E. L. Logan N. Willett B. J. Biek R. Hosie M. J. **(**2011**).** Phylogenetic characterisation of naturally occurring feline immunodeficiency virus in the United Kingdom. Vet Microbiol 150, 239–247. 10.1016/j.vetmic.2011.01.027 21349661 PMC3103826

[r67] Schultz A. K. Zhang M. Leitner T. Kuiken C. Korber B. Morgenstern B. Stanke M. **(**2006**).** A jumping profile Hidden Markov Model and applications to recombination sites in HIV and HCV genomes. BMC Bioinformatics 7, 265. 10.1186/1471-2105-7-265 16716226 PMC1525204

[r68] Seo T.-K. Thorne J. L. Hasegawa M. Kishino H. **(**2002**).** A viral sampling design for testing the molecular clock and for estimating evolutionary rates and divergence times. Bioinformatics 18, 115–123. 10.1093/bioinformatics/18.1.115 11836219

[r69] Shankarappa R. Margolick J. B. Gange S. J. Rodrigo A. G. Upchurch D. Farzadegan H. Gupta P. Rinaldo C. R. Learn G. H. **& other authors (**1999**).** Consistent viral evolutionary changes associated with the progression of human immunodeficiency virus type 1 infection. J Virol 73, 10489–10502. 10559367 10.1128/jvi.73.12.10489-10502.1999 PMC113104

[r70] Shapiro B. Rambaut A. Drummond A. J. **(**2006**).** Choosing appropriate substitution models for the phylogenetic analysis of protein-coding sequences. Mol Biol Evol 23, 7–9. 10.1093/molbev/msj021 16177232

[r71] Sodora D. L. Shpaer E. G. Kitchell B. E. Dow S. W. Hoover E. A. Mullins J. I. **(**1994**).** Identification of three feline immunodeficiency virus (FIV) *env* gene subtypes and comparison of the FIV and human immunodeficiency virus type 1 evolutionary patterns. J Virol 68, 2230–2238. 8139008 10.1128/jvi.68.4.2230-2238.1994 PMC236699

[r72] Tamura K. Peterson D. Peterson N. Stecher G. Nei M. Kumar S. **(**2011**).** mega5: molecular evolutionary genetics analysis using maximum likelihood, evolutionary distance, and maximum parsimony methods. Mol Biol Evol 28, 2731–2739. 10.1093/molbev/msr121 21546353 PMC3203626

[r73] Tebit D. M. Nankya I. Arts E. J. Gao Y. **(**2007**).** HIV diversity, recombination and disease progression: how does fitness “fit” into the puzzle? AIDS Rev 9, 75–87. 17694675

[r74] Teixeira B. M. Logan N. Cruz J. C. M. Reis J. K. P. Brandão P. E. Richtzenhain L. J. Hagiwara M. K. Willett B. J. Hosie M. J. **(**2010**).** Genetic diversity of Brazilian isolates of feline immunodeficiency virus. Arch Virol 155, 379–384. 10.1007/s00705-009-0587-2 20084530

[r75] Terio K. A. Kinsel M. J. Raphael J. Mlengeya T. Lipende I. Kirchhoff C. A. Gilagiza B. Wilson M. L. Kamenya S. **& other authors (**2011**).** Pathologic lesions in chimpanzees (*Pan trogylodytes schweinfurthii*) from Gombe National Park, Tanzania, 2004–2010. J Zoo Wildl Med 42, 597–607. 10.1638/2010-0237.1 22204054 PMC3693847

[r76] Tomasec P. Braud V. M. Rickards C. Powell M. B. McSharry B. P. Gadola S. Cerundolo V. Borysiewicz L. K. McMichael A. J. Wilkinson G. W. **(**2000**).** Surface expression of HLA-E, an inhibitor of natural killer cells, enhanced by human cytomegalovirus gpUL40. Science 287, 1031–1033. 10.1126/science.287.5455.1031 10669413

[r77] Troyer J. L. Vandewoude S. Pecon-Slattery J. McIntosh C. Franklin S. Antunes A. Johnson W. O’Brien S. J. **(**2008**).** FIV cross-species transmission: an evolutionary prospective. Vet Immunol Immunopathol 123, 159–166. 10.1016/j.vetimm.2008.01.023 18299153 PMC2442884

[r78] Ulbrecht M. Martinozzi S. Grzeschik M. Hengel H. Ellwart J. W. Pla M. Weiss E. H. **(**2000**).** Cutting edge: the human cytomegalovirus UL40 gene product contains a ligand for HLA-E and prevents NK cell-mediated lysis. J Immunol 164, 5019–5022. 10.4049/jimmunol.164.10.5019 10799855

[r79] Verschoor E. J. Hulskotte E. G. Ederveen J. Koolen M. J. Horzinek M. C. Rottier P. J. **(**1993**).** Post-translational processing of the feline immunodeficiency virus envelope precursor protein. Virology 193, 433–438. 10.1006/viro.1993.1140 8382405

[r80] Whittle H. Morris J. Todd J. Corrah T. Sabally S. Bangali J. Ngom P. T. Rolfe M. Wilkins A. **(**1994**).** HIV-2-infected patients survive longer than HIV-1-infected patients. AIDS 8, 1617–1620. 10.1097/00002030-199411000-00015 7848600

[r81] Wilgenbusch J. C. Swofford D. **(**2002**).** Inferring evolutionary trees with paup. Curr Protoc Bioinform 6, 6.4. 10.1002/0471250953.bi0604s00 18428704

[r82] Wolfs T. F. de Jong J. J. Van den Berg H. Tijnagel J. M. Krone W. J. Goudsmit J. **(**1990**).** Evolution of sequences encoding the principal neutralization epitope of human immunodeficiency virus 1 is host dependent, rapid, and continuous. Proc Natl Acad Sci U S A 87, 9938–9942. 10.1073/pnas.87.24.9938 1702224 PMC55289

[r83] Yamaguchi Y. Gojobori T. **(**1997**).** Evolutionary mechanisms and population dynamics of the third variable envelope region of HIV within single hosts. Proc Natl Acad Sci U S A 94, 1264–1269. 10.1073/pnas.94.4.1264 9037041 PMC19779

[r84] Zhang L. Diaz R. S. Ho D. D. Mosley J. W. Busch M. P. Mayer A. **(**1997**).** Host-specific driving force in human immunodeficiency virus type 1 evolution *in vivo*. J Virol 71, 2555–2561. 9032400 10.1128/jvi.71.3.2555-2561.1997 PMC191373

